# To avoid operating on pseudo tumoral pulmonary infarctions mimicking lung cancer

**Published:** 2012-05-22

**Authors:** Alexis Lacout, Pierre Yves Marcy, Mostafa El Hajjam

**Affiliations:** 1 Centre d'imagerie Médicale 47 Boulevard du Pont Rouge 15000 Aurillac, France; 2 Head and Neck and Interventional Radiology Department Antoine Lacassagne Cancer Research Institute 33, Avenue Valombrose06189 Nice cedex1 France; 3 Service de Radiologie, Hôpital Ambroise PARE (APHP) 9, Avenue Charles de GAULLE 92100 Boulogne Billancourt France

**Keywords:** Pulmonary embolism, infarction, CT-scan, follow up

## Abstract

Pulmonary infarction usually appears as a hump-shaped triangular opacity with its base applied to a pleural surface. In some cases, pulmonary infarctions may appear as a pseudo tumoral opacity mimicking lung cancer. Thoracotomy could be prevented by repeating CT scan in properly selected patients.

## Introduction

The purpose of this article is to highlight the challenging diagnostic of pulmonary infarction that may mimic lung cancer in order to prevent unnecessary invasive examinations.

## Patient and case report

**Figure 1:**
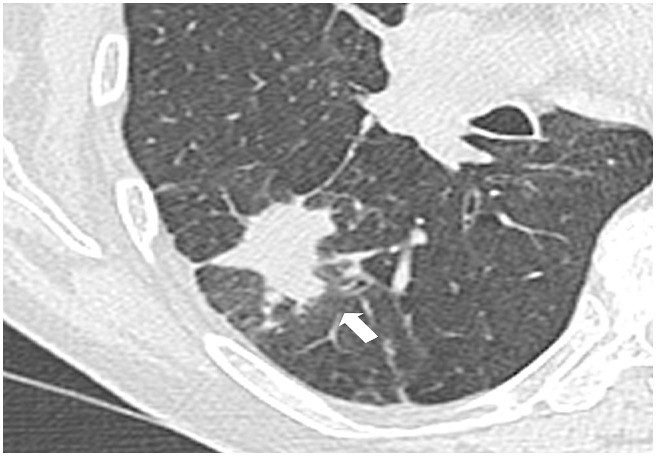
First unenhanced CT scan, with axial reformations, showing a 33-mm spiculated mass of the apical segment of the lower right lobe (large arrow)

**Figure 2:**
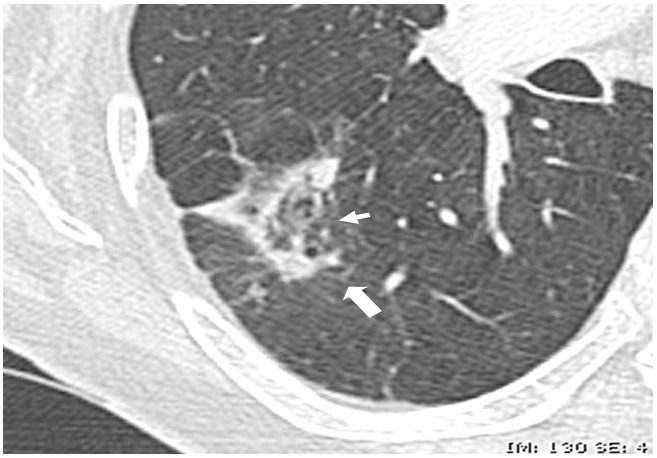
Second post-contrast CT scan, with axial reformations, performed 15 days later showing changes with new central ground glass attenuation (arrow)CT scan examination also showed pulmonary emboli: some clots were central and marginated within the right main pulmonary artery (not shown)

**Figure 3:**
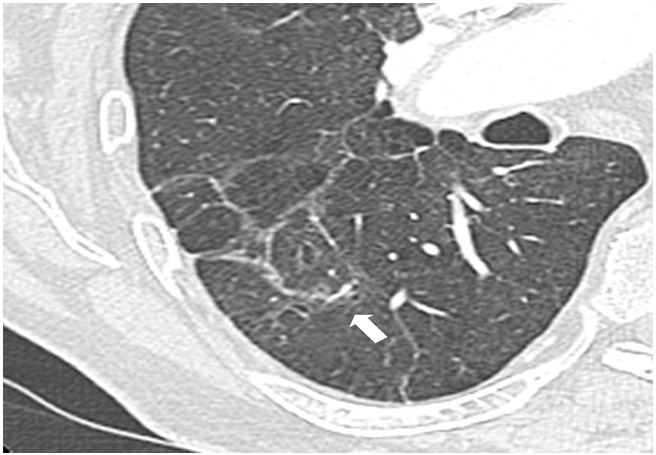
Third post-contrast CT scan, with axial reformation, performed 3 months later showing total disappearance of the mass (large arrow). There was a complete disappearance of pulmonary emboli (not shown)

A 69 year old man who had smoked 50 package-year was referred to our institution to perform a CT scan evaluation of the chest. A first CT scan (Figure 1), performed without contrast, showed a 33-mm spiculated mass of the right lower lobe. The mass was homogenous, located at the lung periphery with pleural contact. Fifteen days later, enhanced CT scan examination (Figure 2) showed significative changes in the mass, namely the appearance of a central ground glass attenuation. CT scan examination also showed pulmonary emboli. Clot margination was in favor of a chronic thrombo embolic event. The patient was then treated with anticoagulant therapy. Three months later, a post-contrast CT scan only showed a residual reticular pattern in place of the initial mass (Figure 3). There was a complete disappearance of pulmonary emboli.

## Discussion

**Figure 4:**
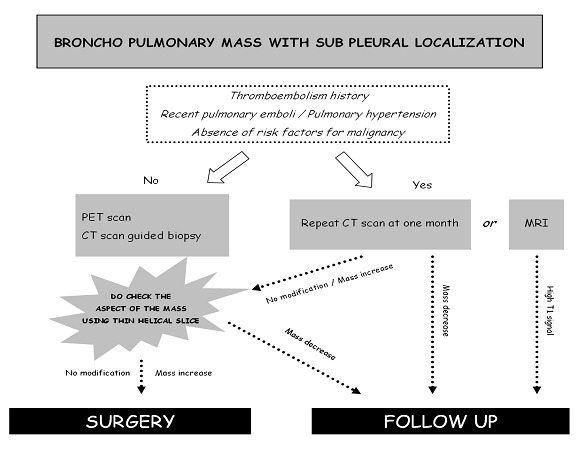
To avoid operating on pseudo tumoral pulmonary infarctions mimicking lung cancer

Consistent with the literature, pulmonary infarction appeared as a pseudo tumoral opacity with nodular, round or irregular shape, usually at the periphery of the lung in a context of thromboembolic disease (Figure 1). The diagnosis of pseudo-tumoral infarction remains a challenge for the following reasons. Pulmonary embolism is not always visualized. PET scan can show increased uptake, and post-contrast CT scan can show enhancement [1]. Additionally, cytology may show pseudo-malignant cells due to cell regeneration induced by pulmonary infarction [1, 2]. Unfortunately, for these reasons many pulmonary infarctions are misdiagnosed for tumors and operated on [3]. On repeat CT scan, the tumor like-mass would disappear over time (Figure 3). Given the large number of lung tumors and the rarity of pseudo-tumoral pulmonary infarctions, watchful waiting cannot be recommended for all pulmonary masses. Elements suggestive of the diagnosis of pulmonary infarction include pulmonary infarction features; i.e. a sub pleural mass of the lower lobes [1], and signs of pulmonary hypertension favorizing the onset of pulmonary infarctions [4]. Absence of risk factor for broncho-pulmonary cancer or a recent thromboembolic event may also be in favor of the diagnosis of pulmonary infarction and may indicate repeat CT scans (Figure 4). When cancer is likely, PET scan or CT-guided biopsy could be performed. Conventional PET scan slices and the tracking slice before CT guided biopsy may be both used to check out the dynamic changes of the mass over time. We stress that millimetric slices (i.e. good spatial resolution) should be used for optimal evaluation of the mass. Mass reduction would suggest to defer more invasive tests and surgical exploration and to prolong watchful imaging-based follow-up. One limitation of this approach is that some pulmonary infarctions may only change slowly over time. In this situation, MRI features may show high T1 weighted signal consistent with alveolar hemorrhage and thus diagnostic of pulmonary infarction [5]. In the other cases, surgical examination may not be avoided, particularly when detersion of the mass is absent, or when mass volume increases.

## Conclusion

Pulmonary infarctions may mimic malignancy. In selected patients, MRI or repeated CT scan may prevent unnecessary thoracotomy.
